# Comparison between contact diode laser with 980 nm and 1470 nm wavelengths for posterior laryngofissure in pigs

**DOI:** 10.1038/s41598-024-62333-3

**Published:** 2024-05-20

**Authors:** Isaac de Faria Soares Rodrigues, Paulo Francisco Guerreiro Cardoso, Natalia Aparecida Nepomuceno da Silva, Aristides Tadeu Correia, Helio Minamoto, Benoit Jacques Bibas, Natalia de Souza Xavier Costa, Marilia Wellichan Mancini, Marisa Dolhnikoff, Paulo Manuel Pego-Fernandes

**Affiliations:** 1 Thoracic Surgery Research Laboratory (LIM-61), Division of Thoracic Surgery, Faculdade de Medicina, Instituto do Coracao, Hospital das Clinicas HCFMUSP, Universidade de Sao Paulo, Sao Paulo, SP Brazil; 2 Departament of Pathology, Faculdade de Medicina, Universidade de Sao Paulo, Sao Paulo, SP Brazil; 3 Department of Biophotonics, Institute of Research and Education in the Health Area (NUPEN), Sao Carlos, SP Brazil

**Keywords:** Medical research, Energy science and technology, Techniques and instrumentation

## Abstract

To compare two different wavelengths of the surgical contact diode laser (CDL) for producing a posterior laryngofissure in in-vivo pigs. Anesthetized pigs underwent a tracheostomy and an anterior laryngofissure through a cervicotomy. They were randomly selected for the CDL wavelength and Power, according to the peak of Power set at device (980nm wavelength: Ppeak power of 10 W, 15 W, and 20 W, or 1470 nm wavelength: Ppeak 3 W, 5 W, 7 W, 10 W). At the end of the experiment, the laryngotracheal specimen was extracted and sent for histology and morphometry measurements (incision size, depth, area, and lateral thermal damage). Hemodynamic data and arterial blood gases were recorded during the incisions. Statistical analysis of the comparisons between the parameters and groups had a level of significance of p < 0.05. Twenty-six pigs were divided into CDL 980 nm (n = 11) and 1470 nm (n = 15). There was a greater incision area at the thyroid level in the 980 nm CDL and a wider incision at the trachea level, with a larger distance between mucosa borders. There were no significant differences in the area of lateral thermal damage between the two groups and neither difference among the power levels tested. Both wavelengths tested showed similar results in the various combinations of power levels without significant differences in the lateral thermal damage. The posterior laryngofissure incision can be performed by either of the wavelengths at low and medium power levels without great difference on lateral thermal damage.

## Introduction

The leading cause of laryngotracheal stenosis (LTS) is orotracheal intubation and tracheostomy^1,2^. The association of laryngeal and tracheal stenosis often requires multiple laborious and technically demanding procedures to achieve a successful outcome. The approach includes an enlargement of the airway diameter through a surgical laryngeal split with costal cartilage graft interposition^3^. Depending on the complexity of the stenosis, the procedure often requires a tracheostomy, placement of a solid silicone stent for 4–6 weeks when it is removed and substituted for a T-tube that remains in place for a longer period to ensure an adequate airway diameter^3^.

The complexity of the laryngotracheal reconstruction combined with the need for long-term stenting harms the patient’s quality of life, with voice and swallowing dysfunction after surgery. Both are related to the extent of surgical trauma followed by local swelling and inflammation that can lead to local scarring.

As an attempt to reduce local surgical trauma, a minimally invasive approach has been proposed using an endoscopic technique and a photonic energy source such as the surgical contact diode laser (CDL)^4,5^. It has been demonstrated experimentally that the open posterior laryngeal split procedure (laryngofissure) can be performed safely and effectively using a 980 nm CDL in pulsed mode with minimal lateral thermal damage to the surrounding tissues if compared to electrocautery, with greater thermal injury^6,7^. Reducing the local tissue trauma can also enhance the healing process, enabling a better and faster functional recovery. Another advantage of the CDL that can reduce the surgical trauma is that the photonic energy is carried by a flexible fiberoptic fiber that allows its endoscopic use in angled surfaces via suspension laryngoscopy, in a minimally invasive approach.

The recent availability and application of the 1470 nm CDL in laryngeal surgery showed its superior tissue penetration without damaging the surrounding tissues^8–10^, therefore suggesting its potential superiority over the 980 nm CDL for upper airway interventions.

This preclinical study compared the 1470 nm CDL versus 980 nm CDL for producing a posterior laryngofissure in in-vivo pigs. The comparison was based on pathological assessment from slices of the airway short time after the experiments and to discover differences between the two wavelengths on measures of the incision and the lateral thermal damage area.

## Materials and methods

The study was approved by the Ethics Committee for Animal Experiments (CEUA 153/14). According to the Brazilian Legislation and the Brazilian College of Animal Experimentation, the animals were treated following the Guide for the Care and Use of Laboratory Animals (Institute of Animal Laboratory Resources–National Academy of Sciences, Washington-USA 1996). The experiments were conducted at the Thoracic Surgery Research Laboratory (LIM 61, InCor-HCFMUSP). Landrace pigs weighing between 15 and 20 kg obtained from the same farm (Giannichi Farm, Sao Paulo- Brazil) were used in all experiments. The animals were sedated with intramuscular injection of 5 mg/kg ketamine (Cetamina, Cristalia, Sao Paulo-Brazil) and 0.5 mg/kg midazolam (Midazolam Teuto Brasileiro, Goias-Brazil), and placed in the supine position in the operating table. An intravenous line was established through dissection of the right internal jugular vein. The intravenous anesthesia started with the injection of a 20 mL bolus of 10 mg/mL propofol (Baxter Pharmaceuticals, India), followed by orotracheal intubation (#6.5 French; Portex, Smiths Medical, Sao Paulo-Brazil), and anesthesia was maintained by a continuous flow (200 mL/min) of inhaled isoflurane (Isoflurano 1 mL/mL, Cristalia, Sao Paulo-Brazil). With the animal in supine position, volume-controlled ventilation started (Mini Ventilador 600, K. Takaoka, Sao Paulo-Brazil; FiO2 1.0, respiratory rate of 20 breaths/minute, tidal volume 10 mL/kg). A right carotid artery was dissected in the neck, and a catheter was inserted and connected to a blood pressure monitor device (Monitor Dixtal, Dx2020, São Paulo-Brazil) for the measurements of mean arterial blood pressure, heart rate, and arterial blood sampling for gas analysis.

A midline neck incision obtained access to the larynx and cervical trachea, and a longitudinal tracheostomy was performed at the level of the 4th tracheal ring. The orotracheal tube was withdrawn, inserted into the tracheostomy, sutured in place, and ventilation resumed with the same parameters. Following the exposure of the larynx by lateral retraction of the strap muscles, an anterior vertical laryngeal split was performed using a #11 scalpel blade (Embramed, Sao Paulo-Brazil), and the animal was randomized to a specific CDL wavelength and power. The distal extremity of the flexible fiber stays in contact with the tissue surface that is aimed to receive the photothermal energy. The posterior laryngofissure consisted of a longitudinal midline incision in the lumen of the posterior wall extending from the glottis and caudally towards the proximal trachea, stopping 1 cm above the tracheostomy. The laryngofissures were performed manually using the CDL (Medilaser Dual 980/1470, DMC, Sao Carlos, SP-Brazil) by the same investigator (IFSR) not blinded for wavelength and potency.

The use of the CDL in pulsed mode was based on its better control of the lateral thermal damage as opposed to the continuous mode (CW)^11–13^. In the pulsed mode, the power experienced by the tissue is the mean power, given by the formula P_mean_ = P_peak_ (W) × pulse width (s) × repetition rate (Hz), where P_peak_ power is the adjusted laser power at the device. The pulsed mode of the laser setup was 1 kHz repetition rate and 500 microseconds pulse width. Thus, the CW power is reduced by the duty cycle factor, given by the pulse width × repetition rate, allowing the tissue to dissipate the absorptive heat generated by the photo-thermal laser-tissue interaction.

The CDL device had dual wavelengths (980 nm and 1470 nm) delivered both individually and simultaneously through the same optical fiber. For the 980 nm wavelength CDL, the P_peak_ power alternated between 10W, 15W, 20W (corresponding to the P_mean_ of 5W, 7.5W, and 10W, respectively). For the 1470 nm wavelength CDL, the P_peak_ power alternated between 3W, 5W, 7W, 10W (corresponding to the P_mean_ of 1.5 W, 2.5W, 3.5W, 5W, respectively). P_mean_ represents the actual power experienced by exposed tissues in the pulsed mode. The laser energy carrier was a biocompatible 400 μm core silica optical fiber (FO 400 Model B, DMC Laser, Sao Carlos, SP-Brazil). During the laser activation, the research team wore protection goggles with filter blocking (Model DMC-02, DMC, Sao Carlos, SP-Brazil).

Arterial blood samples were drawn from the carotid artery catheter at the end of the experiment following the extraction of the larynx and trachea before sacrifice. The samples were sent for pH, lactate, hematocrit, and arterial blood gas analysis. The heart rate (HR) and mean arterial blood pressure (MAP) recordings were obtained at the beginning of the cervicotomy, during the laryngofissure of the posterior wall of the airway, and after the laryngotracheal extraction before sacrifice. Immediately after the extraction and under general anesthesia, the animal was sacrificed by an intravenous bolus injection of 19.1% potassium chloride, 0.3 mL/kg (Isofarma, Ceara-Brazil) as the local Ethics Committee for Animal Experiments recommended.

**Figure 1:**
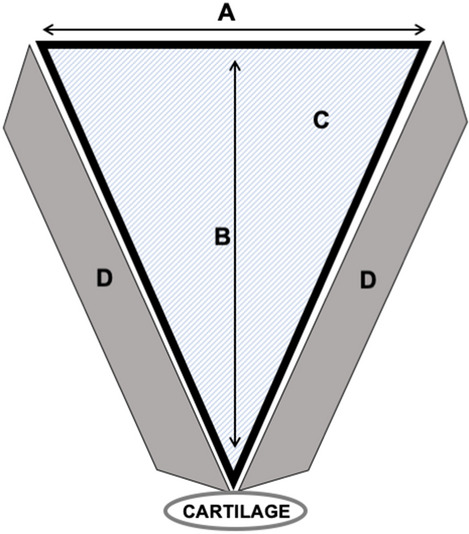
Morphometry measurements for the histopathological analysis: (**A**) distance between the cut margins of the incision, on top between the superficial tissue of intact mucosa; (**B**) distance from the top line of the cut margin of previous mucosa to the bottom of the incision; (**C**) area of the crater of the incision; (**D**) area of the lateral thermal damage in the surroundings.

The larynx and upper third of the trachea were resected *en bloc* and immersed in 10% formaldehyde (Anidrol, Sao Paulo-Brazil) for histopathology. The specimens were sliced transversely in the pathology laboratory and separated according to the following levels: Larynx (from the thyroid to the cricothyroid membrane); Cricoid (from the upper margin of the cricoid cartilage to the upper margin of the 1st tracheal ring); Trachea (from the 1st to the 5th tracheal ring). The separated specimens were embedded in paraffin, routinely processed, and stained with hematoxylin–eosin (Hematoxylin—Sigma-Aldrich, St. Louis, Missouri, USA; Eosin—Labsynth, Sao Paulo-Brazil). The slides were then scanned digitally (Pannoramic P250 Flashdigital, 3DHISTECH Ltd, Budapest, Hungary) using a 40 × power magnification objective (Plan-Apochromat, Zeiss, Jena, Germany) attached to a CIS color camera (CIS Corporation, Tokyo, Japan) for bright field image acquisition. The images obtained underwent morphometry measurements using a Case Viewer (3DHistech, Budapest, Hungary) and were assessed by one investigator (I.F.S.R.) trained and supervised by two experienced pathologists (M.D.; N.S.X.C.). The measurements evaluated in this study consist into the following (Fig. 1):

- Distance A consists in the width of the crater made by the incision at the top part, separating the borders of intact mucosa;
- Distance B is the depth of the crater, of a perpendicular line from the measure A to the bottom where remains the deep layer of tissue.

Those two first distance were measured in micrometers (µm).

- The area C represents the total area of the crater created after the incision at that sliced picture. This area was delimited by measure A and the internal surface of tissue inside the crater.
- The area D was the collateral thermal damage in the surroundings, specially on the mucosa and submucosa. It was drowned after electronic microscopy assessment of coagulated or necrotic tissue.

The two areas were measured in square micrometers (µm^2^).

**Figure 2:**
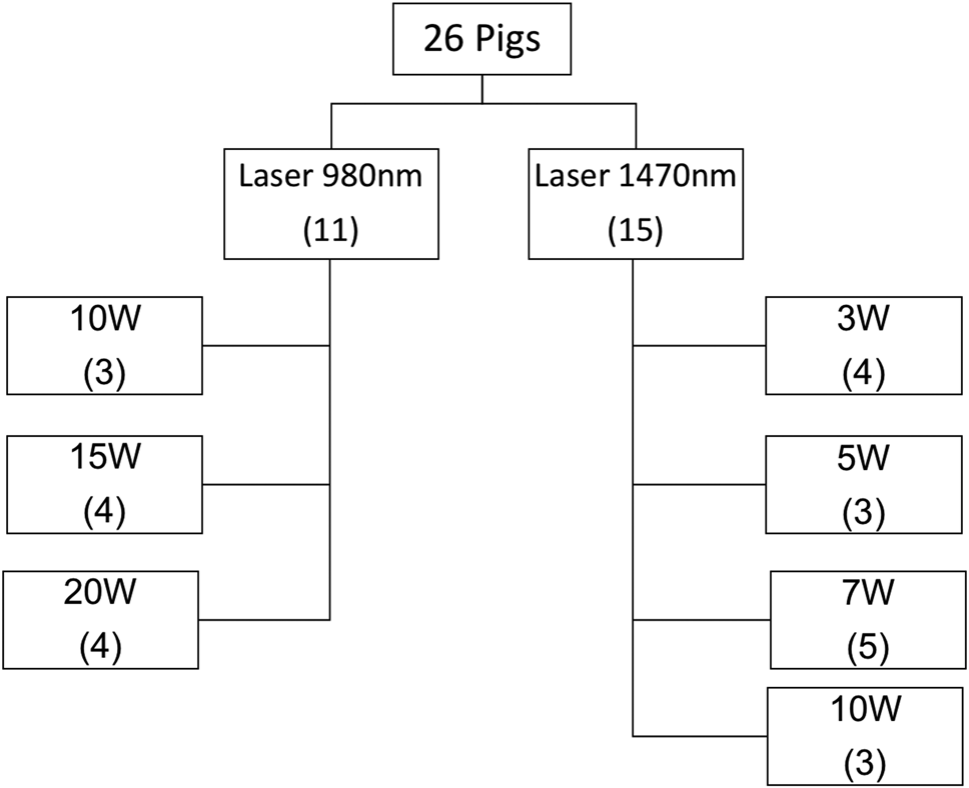
Distribution of the animals according to group (980 nm CDL vs. 1470 nm CDL), and to the following power levels: P_peak_ 980 nm group (10W, 15W, 20W), corresponding to P_mean_ of 5W, 7.5W, 10W, respectively; P_peak_ 1470 nm group (3W, 5W, 7W, 10W), corresponding to P_mean_ of 2.5W, 3.5W, 5W, respectively.

Twenty-six landrace pigs of both sexes (mean weight 17.4 ± 2.7 kg) were randomly assigned to the 980 nm CDL group (n = 11), and to the 1470 nm CDL group (n = 15), with their respective potencies (Fig. 2).

### Statistical analysis

The data is presented as mean and standard error of the mean. Normally distributed data in each group and homogeneity of the variances between groups were assessed using the Shapiro–Wilk and Levene tests. Comparisons between mean values used the One Factor Variance Analysis, whereas two parameters (type of energy and power level) used double factor analysis of variance. The analyses were performed using the SPSS 21 computer software for Windows (IBM, Armonk, New York, USA), and the significance level was set for p < 0.05.

### Ethical approval

The Study was realized according to ARRIVE guidelines (Animal Research: Reporting of In Vivo Experiments), Ethics Committee for Animal Experiments (CEUA 153/14). According to the Brazilian Legislation and the Brazilian College of Animal Experimentation.

## Results

There were no significant differences between the male and female groups with the wavelength of the energy delivered (p = 0.127). There were no significant differences in body weight, hematocrit and arterial blood O_2_ saturation between the groups, in a way that the water proportion at the tissue or the concentration of hemoglobin would not interfere favoring either 1470 nm or 980 nm. The PaO_2_ was higher in the 980 nm group, and the lactate values were higher in the 1470 nm group. The heart rate was not different between the study groups, as were the doses of anesthetic drugs used in all animals. The duration of the incisions consisted the timed period from the start of incision with the laser until the end, using a stopwatch (TS-1809, Taksun Quartz Timer, China). It was longer in the 1470 nm group when compared to the 980 nm group (p = 0.02) (Table 1).

**Table 1:** Hemodynamic and blood gases data.

Parameter	980 nm	1470 nm	*p*
Body weight (kg)	18.9 ± 3.30	17.8 ± 1.00	0.348
Mean arterial pressure (mmHg)	59.5 ± 8.49	64.5 ± 9.67	0.185
Heart rate (beats/minute)	115.4 ± 15.02	125.2 ± 16.07	0.126
pH	7.5 ± 0.17	7.5 ± 0.17	0.432
PaO_2_ (mmHg)	422.5 ± 43.18	351.8 ± 115.94	0.039
PaCO_2_ (mmHg)	18.6 ± 4.52	18.6 ± 3.0	0.980
O_2_ saturation (%)	99.7 ± 0.5	99.2 ± 1.19	0.254
Hematocrit (%)	32.7 ± 5.07	31.4 ± 3.64	0.439
Lactate (mmol/L)	1.9 ± 0.72	3.1 ± 1.80	0.030
Incision duration (seconds)	77.8 ± 35.63	188.5 ± 107.86	0.02

**Figure 3:**
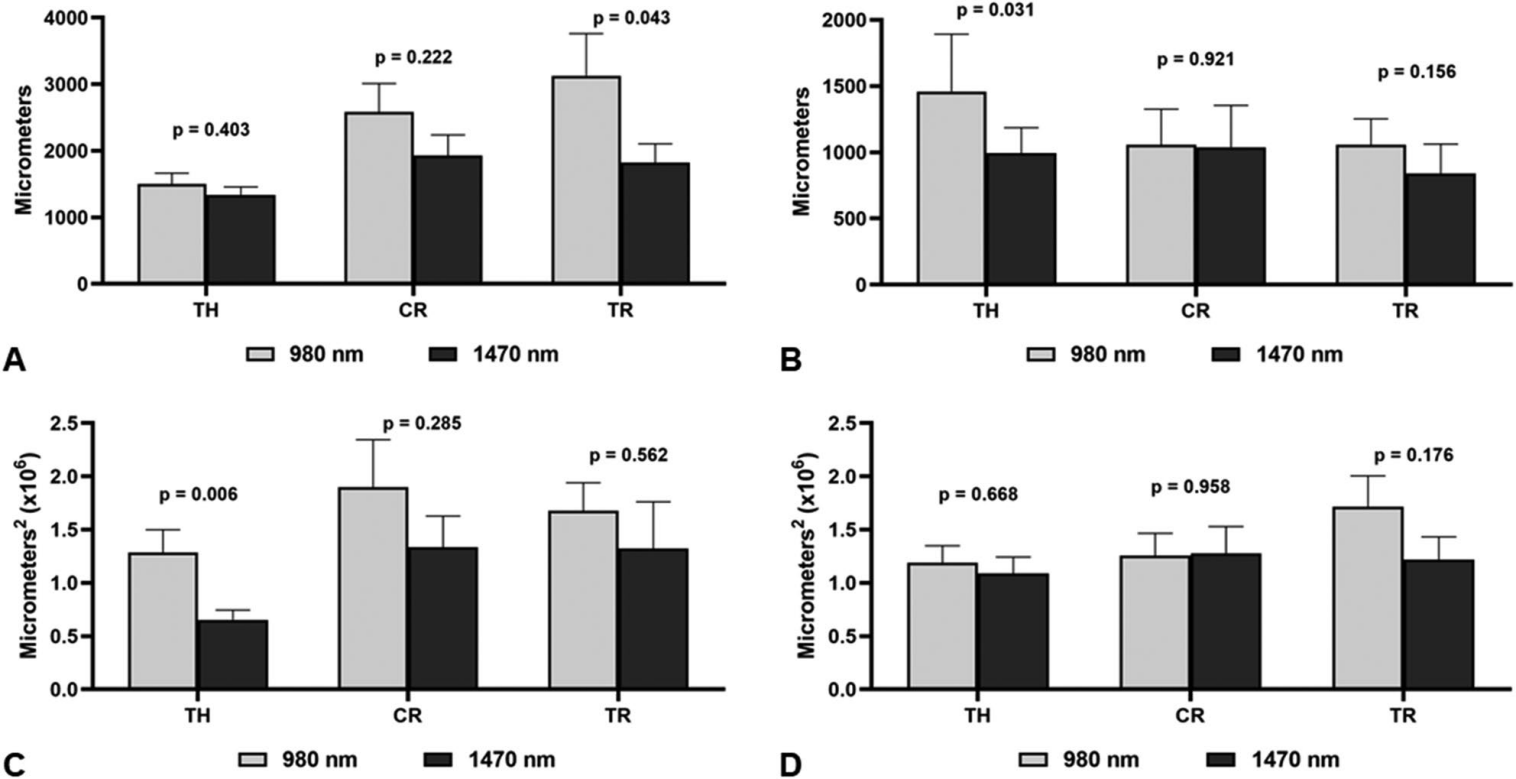
Comparison of the morphometry measurements between the 980 nm CDL group and the 1470 nm CDL group. (**A**) Measure A: distance between top borders of cut mucosa. (**B**) Measure B: Depht of incision. (**C**) Measure C: Area produced by the incision. (**D**) Measure D: Lateral area of thermal damage.

The measurements of the incisions at different P_peak_ 1470 nm group—3W, 5W, 7W, 10W (corresponding to P_mean_ of 2.5W, 3.5W, 5W); P_peak_ 980 nm group -10W, 15W, 20W (corresponding to P_mean_ of 5W, 7.5W, 10W) in the 3 locations (thyroid cartilage, cricoid cartilage, and trachea) are described in Fig. 3. In the 1470 nm CDL group, the distance between the borders of the mucosa (measurement A) was significantly smaller in the trachea, and deeper at the level of the thyroid cartilage (measurement B). The total area of the incision was smaller in the 1470 nm CDL group at the level of the thyroid cartilage (measurement C). No significant differences were found between the groups in the lateral thermal damage area (Fig. 3).

**Figure 4:**
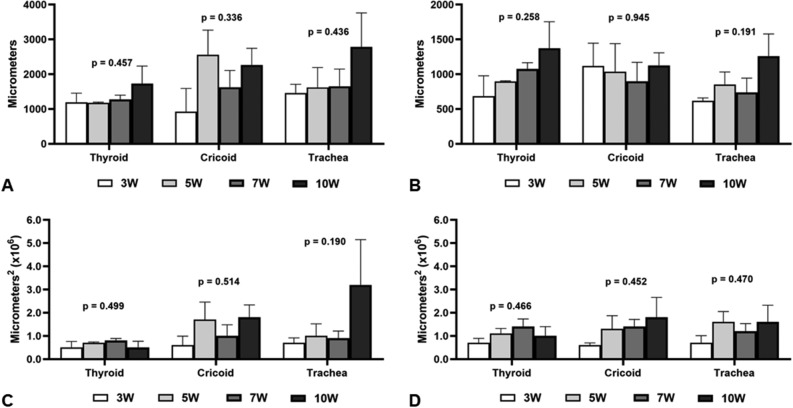
Comparison of the power levels used in all 1470 nm CDL group locations. P_peak_ 1470 nm group—3W, 5W, 7W, 10W (P_mean_ of 2.5W, 3.5W, 5W, respectively). (**A**) Measure A: distance between top borders of cut mucosa. (**B**) Measure B: Depht of incision. (**C**) Measure C: Area produced by the incision. (**D**) Measure D: Lateral area of thermal damage.

A separate analysis of the 1470 nm CDL group showed no differences in thermal damage area in the different locations of the incisions when all potencies were compared (Fig. 4).

**Figure 5:**
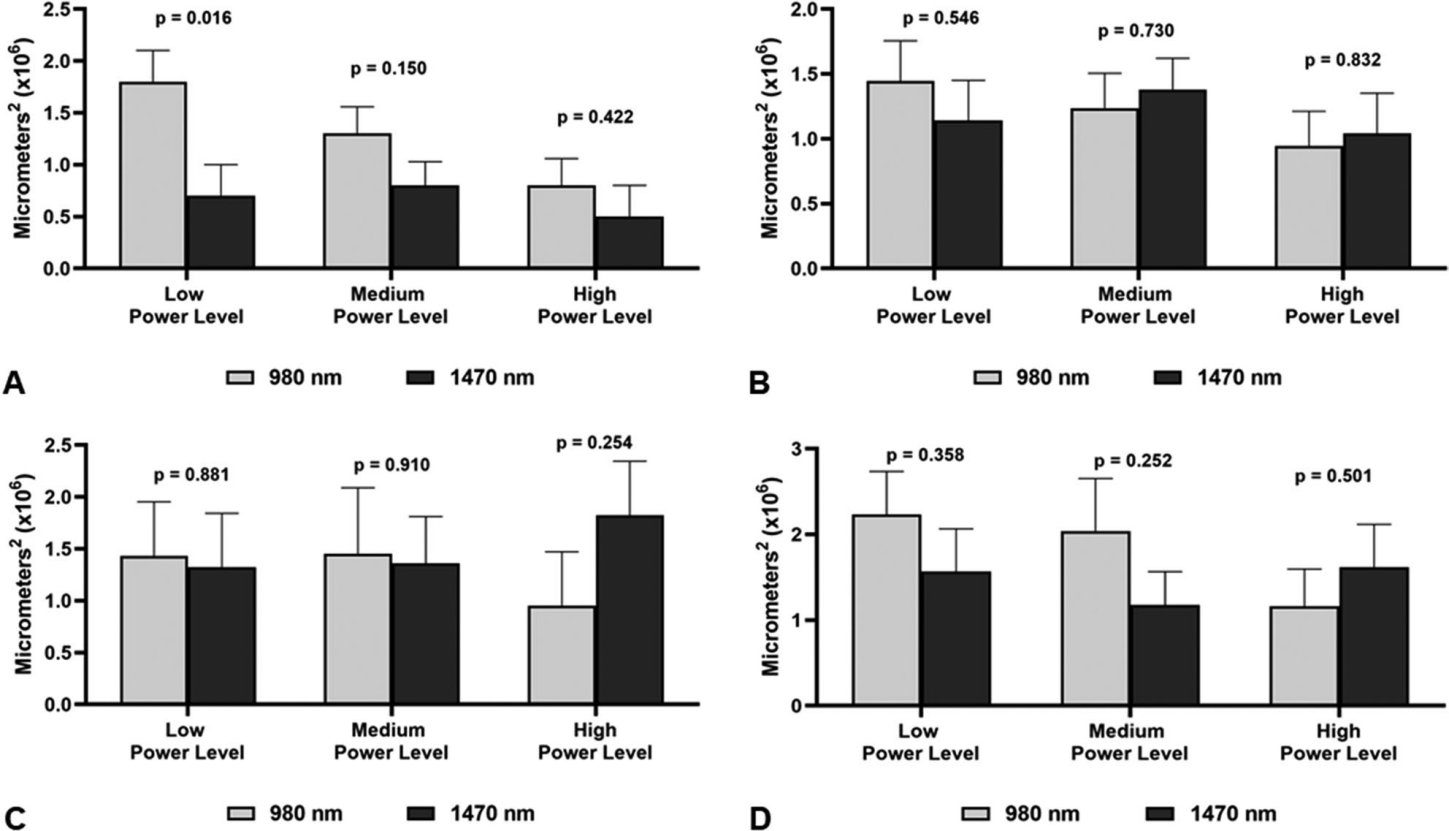
Comparison of power levels according to subgroups Low, Medium and High Power levels. (**A**) Measure A: distance between top borders of cut mucosa. (**B**) Measure B: Depht of incision. (**C**) Measure C: Area produced by the incision. (**D**) Measure D: Lateral area of thermal damage.

The 3W experiments in the 1470 nm group were excluded from the analysis because of their very low power density, which resulted in almost no detectable tissue impact. For subgroup analysis, the power levels used were grouped into three power levels: Low power level (P_peak_ = 5W for the 1470 nm CDL group and 10W for the 980 nm CDL group); Medium power level (P_peak_ = 7W for the 1470 nm CDL group and 15W for the 980 nm CDL group); High power level (P_peak_ = 10W for the 1470 nm CDL group and 20W for the 980 nm CDL group). The only significant difference found was the larger incision area in 980 nm produced with the lower power level at the thyroid cartilage (Fig. 5A). The thermal damage area was not different between 980 and 1470 nm CDL incisions at all power levels, and across the different anatomic areas where incisions were made (Fig. 5B–D).

## Discussion

The LTS presents as dense fibrous scar tissue extending from the posterior cricoid to the subglottis and to the proximal trachea^1^. Treatment of LTS often requires multiple procedures such as dilatation, stenting, and surgery. Surgical treatment of LTS includes a combination of the resection of scar tissue and laryngotracheal enlargement of the airway lumen at the posterior cricoid level using a posterior cricoid split with costal cartilage interposition^2,3^. Laryngotracheal resection and reconstruction is more more surgical than standard cervical tracheal resection. Therefore, it has more post-operative complications, such as wound infection and dysphonia^3^. The recovery after laryngotracheal resection and reconstruction is also slower due to the extensive local surgical tissue manipulation. Depending on the extent of the LTS and the severity of the airway narrowing, there is the need for a tracheostomy or prolonged post-operative airway stenting to secure the airway diameter until healing is completed and decannulation is deemed feasible, which may take as long as 1 to 3 years^3^. Many patients requires speech therapy to manage post-laryngotracheal surgery voice and swallowing disturbances often associated with the open surgical laryngeal split^4^.

The rationale for developing an endoscopic cricoid split in substitution for the open surgical approach in LTS focuses on reducing local surgical tissue trauma and less post-operative morbidity that may yield a faster recovery and a better laryngeal function. In this scenario, a surgical contact laser has been considered to perform the posterior cricoid split because it provides coagulation and a more precise cutting with minor tissue swelling, resulting in a faster and painless post-operative recovery^14^. Clinical experience with dental surgery has shown the advantages of lasers over the scalpel for soft tissue surgery including decontamination of the surgical area, less postoperative bleeding, reduced inflammatory response, and less postoperative pain^15^.

The 980 nm CDL is safe and effective for the treatment of laryngeal and upper airway diseases such as stomal granulomas, vocal polyp, glottic web, papilloma, glottic carcinoma, bilateral cord palsy, subglottic cyst or airway stenosis. Each situation will dictate the laser parameters and the expected effects, such as vaporization, cutting, or coagulation^8^. The flexible fiber optic allows it to bend up to a 60-degree angle towards the posterior wall of the airway, facilitating its use through a suspension laryngoscope. Furthermore, it may obviate the need for a surgical anterior thyroid cartilage split^16^.

A previous study showed the feasibility, safety, and effectiveness of the 980 nm CDL carried by flexible fiber optics in the power range of P_peak_ of 10W to 20W (P_mean_ 5W and 10W, respectively) for posterior laryngeal split^6^. The energy absorption of the 980 nm CDL is centered in the hemoglobin. Hence, the coagulation time increases and reaches the ablation temperature after photonic energy has been delivered^9,10^. Conversely, in the 1470 nm CDL, most laser light absorption is by water, whereas the hemoglobin absorption is negligible. In human tissues, the photo-thermal interaction offers both ablative and coagulative tissue effects simultaneously^17,18^. Therefore, even at low power, the 1470 nm CDL has a photo-thermal interaction capable of delivering enough energy to be converted into ablation and coagulation of the surrounding tissues.

The present study compared the 1470 nm CDL and the 980 nm CDL wavelength for the posterior laryngeal split. The inclusion of the hemodynamic measurements, hematocrit, and gas exchange during use of the CDL showed that upper airway perfusion was not affected during the laser incisions. So the flow and amount of water tissue would not be a bias into this analysis. The differences found in the physiologic parameters between the groups were restricted to the arterial blood lactate levels (higher in the 1470 nm group), and PaO_2_ (higher in the 980 nm CDL group). Nevertheless, in the 1470 nm CDL it remained within the hyperoxic level (so neither a reduction in water tissue nor in hemoglobin concentration). The energy deposition would impact directly into one of the outcomes of the study: the lateral area affected by coagulation or necrosis from the thermal destruction. The energy deposition converted into heat had some variables: the intensity of power level of the delivered, and for how long the energy would be applied. Higher power level or longer durations would be greater accumulation of heat and thus, greater dispersion and coagulation effect.

The previous study showed that the 980 nm CDL caused less lateral thermal damage than electrocautery. However, the present study’s comparison between incisions produced by the 980 nm and 1470 nm CDL showed no significant differences in the lateral thermal damage. The stratification into low, medium, and high-power levels was also tested between the two wavelengths at the three anatomical regions (thyroid, cricoid, and trachea). The differences found were limited to the smaller incision area at the thyroid level in the 1470 nm CDL at low power level, whichi could be explained by the very low intensity of the 1470 nm CDL (P_peak_ 5W; P_mean_ 2.5W) compared to the 980 nm CDL (P_peak_ 10W; P_mean_ 5W).

The tissue perfusion interferes with the photo-thermal tissue effects. Janda et al.^19^ demonstrad in ex vivo animal muscle that both Nd:YAG laser and CDL cause a lower heating effect when used in deep incisions. Using the diode laser in contact mode causes, more carbonization on the tissue surface with smaller neighboring areas of coagulation. Smaller wavelengths, such as 830 nm, cause wider ablation, whereas 830 nm and 940 nm yield to severe carbonization in high-power levels (10W and 20W) either in continuous or pulsed mode. The authors suggested using low power levels to reduce carbonization and improve results.

The power settings used for the 980 nm CDL and 1470 nm CDL derived from preliminary studies^6,8,19–21^. Theoretically, the 1470 nm CDL at low power outperforms the 980 nm CDL because its absorption coefficient (μ_A_) by water molecules of soft tissue chromophores is 50 times higher than 980 nm CDL. The high water μ_A_ of the 1470 nm CDL also results in a shallow optical penetration depth (μ_A_ = 1/μ_A_) of the photons compared to the 980 nm CDL^22,23^. The absorptive heat level Q (J/mm^3^) also depends on μ_A,_ so this interaction of the energy penetration from the 1470 nm being slighter than other wavelengths results in lesser heat dispersion and accumulation. The mechanism of thermal dissipation of the heat by the tissue also plays a role in the total energy spread and in the extent of thermal lateral damage.

The photothermal interaction caused by the CDL with the tissue involves several biological effects according to the generated heat in situ and the subsequent local temperature rise. Hyperthermia starts at temperatures of 43 °C to 45 °C and is reversible. When the tissue temperature reaches 60 °C to 65 °C there is protein denaturation along with the coagulation of blood vessels resulting in tissue necrosis. Temperatures exceeding 100 °C yield to protein breakdown with the release of hydrogen, nitrogen, and oxygen, leaving a layer of carbonization behind. The degree of such changes depends on the laser–tissue interaction parameters^13^. Increasing the laser power settings does not necessarily result in a linear increase of the thermal effect. The temperature rise after heat accumulation also influences the optical, thermal and the mechanical tissue parameters^19^. This knowledge is one of the cornerstone bases when deciding when, why and how apply a laser delivering heat into a well-perfused tissue such as the airway mucosa. In the later outcome excessive coagulation or necrosis would turn into excessive and uncontrolled fibrosis and scarring.

Most of the laser light is absorbed within the first 60 μm of the tissue depth. High power and the continuous mode often led to heat accumulation and more thermal damage to adjacent structures^22–25^. The lower larynx and subglottis have thick mucosa, therefore with more tissue water, resulting in greater energy dispersion and coagulation^26^. In the present study, the incisions made by the 1470 nm CDL resulted in smaller incision area and depth in the thyroid area, possibly derived from a better performance of the 1470 nm CDL compared to the 980 nm CDL at this site. Even without significant and homogenous difference at all places, the smaller measures of 1470 nm conceivably related to the this wavelength water properties already depicted. Something similar occurred with a shorter distance between the borders of the incision on the mucosa at the trachea with the 1470 nm CDL.

The comparison between the 3 power levels showed that the larger incision area at the thyroid level produced by the 980 nm CDL at low power levels was associated with a higher intensity (10W) than the 1470 nm CDL at 5W.

The incision shape and width are mode-dependent (continuous/pulsed), whereas lateral thermal damage results from complex interactions between different laser parameters^27^. Nevertheless, the common denominator is that most laser-tissue interactions can produce some degree of tissue vaporization and a surrounding area of thermal necrosis^15^. In addition to the tissue necrosis and water contents, would be other factors causing impact on the extension of thermal damage, such as the manual handling of an operator, the patient movement^28^, as well as the speed and the local pressure applied to the CDL to produce the incision. Beer et al. showed that the area and depth of carbonization and necrosis in CDL incisions are correlated with the cutting speed. The area and depth of the reversible damage are linked to the average power used^12^. The wound repair that follows the incisions is related to the individual variability in handling the device^11^.

The limitations of this study are mostly related to its experimental design that restricts the assessment of the incisions to the short term. Nevertheless, we believe these findings in swine can be transposed to humans because of the inter-species similarities, particularly regarding the upper airway anatomy, histology, and blood rheology. We also acknowledge that the major technical limitation was the manual performance of the incisions. The time needed to execute the incisions manually varied and was longer in the 1470 nm CDL group. The surgeon (I.F.S.R.) identified the 980 nm CDL’s deeper incisions and the shorter time needed to perform the incisions in the posterior laryngotracheal mucosa, particularly at high-power levels. It also explains the longer time required to produce the same macroscopic aspect of the incision produced by the 1470 nm CDL. Such issues can be dealt by using robotically assisted incision. Another issue considered is the time required to produce the incision and its effect on the lateral thermal damage. A longer irradiation time is often related to deeper thermal depths^29^. Lastly, the sort-term character of this study does not allow conclusions regarding the long-term outcomes of the incisions.

## Conclusion

The present study showed that CDL wavelengths tested produced similar results. As a result of its coagulative and heat dispersion properties, the 1470 nm CDL produced smaller lateral thermal damage at a lower and medium power levels and slightly more significant lateral thermal damage at high power levels, but none have reached a statistically significant difference. The posterior laryngofissure incision with CDL can be effectively performed with either wavelength. The best result regarding tissue interaction with the CDL is achieved with the settings at low and medium power levels.

## Data Availability

The data recorded and analyzed during the current study are stored with the authors at InCor and available from the corresponding author at reasonable request.
